# Comparative Investigations on Properties of Three Kinds of FDM 3D-Printed Natural Plant Powder/Poly(lactic acid) Biocomposites

**DOI:** 10.3390/polym15030557

**Published:** 2023-01-21

**Authors:** Dezhi Xu, Jianan Shi, Rui Qiu, Wen Lei, Wangwang Yu

**Affiliations:** 1 College of Science, Nanjing Forestry University, Nanjing 210037, China; 2 Organization Department of the Party Committee, Nanjing Vocational University of Industry Technology, Nanjing 210023, China; 3 School of Mechanical Engineering, Nanjing Vocational University of Industry Technology, Nanjing 210023, China

**Keywords:** poly(lactic acid) (PLA), astragalus residue powder (ARP), wood flour (WF), rice straw powder (RSP), biocomposite, fused deposition modeling (FDM)

## Abstract

In order to further explore the feasibility of the application of the residue of Chinese herbal medicine in FDM 3D technology and enrich the kinds of printing materials, Astragalus residue powder(ARP)/poly(lactic acid) (PLA) biocomposite was FDM 3D-printed, meanwhile, two traditional biocomposites, i.e., wood flour (WF)/PLA and rice straw powder (RSP)/PLA, were prepared by the same method, and the properties of the biocomposites were comparatively investigated. The results showed that, the tensile and flexural strengths of ARP/PLA were 28.33 MPa and 97.60 MPa, respectively, which were 2.85% and 10.89% smaller than those of WF/PLA, while 15.73% and 7.04% greater than those of RSP/PLA. WF/PLA showed typical brittle fracture characteristics, ARP/PLA and RSP/PLA both showed ductile fracture, but not obviously. Among the three kinds of biocomposites, ARP/PLA was the most thermally stable, followed by WF/PLA and RSP/PLA in turn. The incorporation of natural plant powder had no significant effect on the glassy transition, melting, and cold-crystallization behaviors of PLA, but the crystallinity of PLA could be increased from 0.3% to 2.0% and 1.9%, respectively, by adding ARP and WF. At 20 °C, the storage modulus of ARP/PLA, WF/PLA and RSP/PLA was 2759.4 MPa, 3361.3 MPa, and 2691.5 MPa, respectively, indicating that WF/PLA has the greatest stiffness, and the stiffness of RSP/PLA was the least. In addition to these, all the biocomposites were hydrophilic, the contact angle of the distilled water on the surface of ARP/PLA, WF/PLA or RSP/PLA was correspondingly 73.5°, 77.6° and 71.2°. Overall, it can be concluded that ARP/PLA has moderate strengths, stiffness and wettability, meanwhile, it is the most thermal stable among the three biocomposites, and can be processed at a temperature close to that of PLA. ARP/PLA is suitable as a new kind of feedstock material for FDM 3D printing.

## 1. Introduction

Three dimensional (3D) printing, also known as additive manufacturing (AM), is a rapidly evolving digital technology that integrates digital molding, mechanical and electrical control, information technology, material science, chemistry and many other fields of advanced technology [1,2], which produces 3D objects for a variety of applications such as works of art, teaching models, lamp frames, and pen containers by manner of layers [3]. Among 3D printing technologies, the additive manufacturing of fused deposition modeling (FDM) is currently the most widespread 3D printing method [4,5], which allows the fabrication of complex parts from a three-dimensional computer model of the part, without requiring a mold. Its design-to-manufacturing cycle is very short, and very little material may be wasted during production. In the FDM process, a polymeric filament is continuously extruded and deposited on a platform on a layer-by-layer basis according to the cross-sectional geometry of the component until a 3D part is formed [6,7].

Polylactic acid (PLA), a highly versatile aliphatic polyester derived from 100% renewable resources, is a biodegradable semi-crystalline biomaterial with decent biocompatibility, good melt processability, compostability, and reproducibility, which can be degraded by both H_2_O and CO_2_ [8] and is more environmentally friendly as compared to the petroleum plastics such as polypropylene, polyethylene, and acrylonitrile butadiene styrene (ABS) [9,10]; its low thermal expansion coefficient makes it resistant to warping, which contributes to dimensional accuracy, and as a result of this, PLA has been explored as a feedstock material for FDM 3D printing in numerous research works [9]. However, its high price limits the wider application of PLA’s FDM technology.

Natural fibers are increasingly used as reinforcements for thermoplastic composites [11,12,13]. PLA-reinforced with natural fibers (such as wood [14], sugarcane bagasse [15], pineapple leaf fiber [16], bamboo [17], flax [17], rice husk [18], kenaf [19], and jute [20]) not only possess a promising range of specific mechanical properties in combination with a reduced environmental footprint, but can also greatly reduce the cost of 3D printing materials, a number of experimental investigations have thus been conducted focusing on the development and characterization of new natural fiber/PLA biocomposites, among which 3D-printed wood flour (WF)/PLA biocomposites and rice straw powder (RSP)/PLA biocomposites have been documented in numerous studies, for example, Ehsan et al. [21] produced WF/PLA filaments and then 3D-printed architected cellular composites. The experimental results demonstrated that the properties of PLA, such as the toughness, the thermal conductivity, and the fracture strain could obviously be increased. Nadir Ayrilmis et al. [22] investigated the effects of the wood flour content on surface properties of 3D-printed materials produced from the WF/PLA filament, which found that the incorporation of WF into the PLA filament increased the surface roughness and significantly decreased the wettability of the specimens. Guo et al. [23] fabricated the WF/PLA biocomposites with FDM and discussed the effect of toughening agents on the properties of the printed specimens, and the impact strength of the biocomposites were increased by 51.31% when 10 wt% thermoplastic polyurethane as the toughening agent was used. Yu et al. [24] developed a new kind of environmentally friendly composite filament for FDM 3D printing based on RSP/PLA biocomposites, exploring the effects of the RSP size and pretreatment on the properties of the printed samples, when RSP was synergistically pretreated by alkaline and ultrasound, the composite exhibited the increased mechanical properties, thermal stability, and hydrophobic properties than untreated RSP/PLA. Yu et al. [25] also experimented the effects of the soil burial of rice straw on the morphology and properties of 3D-printed RSP/PLA biocomposites, and they concluded that, more cavities with large holes could be observed on the surfaces of the samples with the extension of soil burial time of rice straw. As a result, the printed samples became more thermally stable when using buried rice straw while their mechanical properties became poorer due to the amplification of the cavities. The crystallinity of the samples was all lower than 9% regardless of whether the rice straw was buried.

To date, the residue of Chinese herbal medicine to be used for FDM 3D technology has seldom been reported. As one of the most important Chinese herbal medicines, Astragalus has been widely grown in China, however, it is always disposed after drug component extraction by cooking. However, the main components of Astragalus are not changed after cooking; in this situation, it can be used as one of the natural fibers in the manufacturing of composites, in addition to the composites with the residue of Astragalus (ARP) which might have some medical effects on the environment. Based on these considerations, we once FDM 3D-printed ARP/PLA biocomposites and investigated the effects of the printing parameters on the performances of the samples in our previous manuscript [26], and it was found that the specimens had proper comprehensive properties.

In order to make the properties of the FDM 3D-printed ARP/PLA biocomposites clearer, as a continuation of previous work, the properties of the products were comparatively investigated with those of FDM 3D-printed WF/PLA and RSP/PLA composite specimens in this article, aiming to develop various environmentally friendly printing materials for FDM technology.

## 2. Experimental

### 2.1. Materials

**Figure 1:**
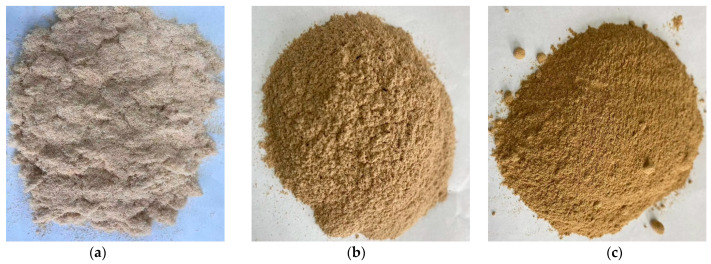
The real photos of different natural fiber powders (**a**) ARP; (**b**) RSP; and (**c**) WF.

PLA (American Nature Works Co.,3052D, Minnetonka, MN, USA) was obtained from the Shanghai Xingyun International Trade Co., Ltd., China (Shanghai, China); the root of Astragalus membranaceus (Fisch.) Bge, which had been grown in the Shanxi province of China for approximately 2 years, was washed, then the drug component was extracted by cooking, after which the root was cooled to room temperature and ground into ARP; the stem of rice straw, which had been grown in the Jiangsu province of China for approximately 6 months, was washed, dried, and then ground into RSP; poplar trunk, which was grown in the Jiangsu province of China for approximately 5 years, was sawn and the sawdust was collected as WF; the average sizes of ARP, WF, and RSP used in this research are all 125 μm. The visual appearances of the natural plant powders were exhibited in Figure 1.

### 2.2. Extrusion of Composite Filaments

To FDM 3D print natural fiber/PLA biocomposite, the cost and the quality of the filament should both be taken into consideration. It was well known that an increased dosage of natural fiber will reduce the cost the filament, while it was found that the flow ability of the melt became poorer when more natural fiber was used, especially for the ARP/PLA biocomposite, as it would be much more difficult to extrude the melt out of the die and obtain a qualified filament when the dosage of ARP was more than 11%-wt. Thus, the dosage of each natural fiber powder was chosen as 11%-wt. in this research.

ARP, RSP, and WF were dried for 8 h at 80 °C before the extrusion to remove moisture, and then three formulations were prepared, respectively: PLA with 11%-wt ARP; PLA with 11%-wt RSP; and PLA with 11 %-wt WF. All batches were compounded with a twin screw extruder (SHJ-20, Nanjing Giant Machinery Co., Ltd., Nanjing, China) at 20 rpm and 130~160 °C for PLA composites, the extruded composites were then granulated to make pellets. To produce the filament, a twin-screw extruder (KS-HXY, Kunshan Huanxinyang Electrical Equipment Co., Ltd., Suzhou, China) was used with the process conditions of 20 rpm and at a temperature between 170 °C and 190 °C from hopper to die. Filaments with a diameter of 1.75 ± 0.05 mm were achieved by using a 2 mm diameter nozzle.

### 2.3. 3D Printing Pieces by FDM

**Figure 2:**
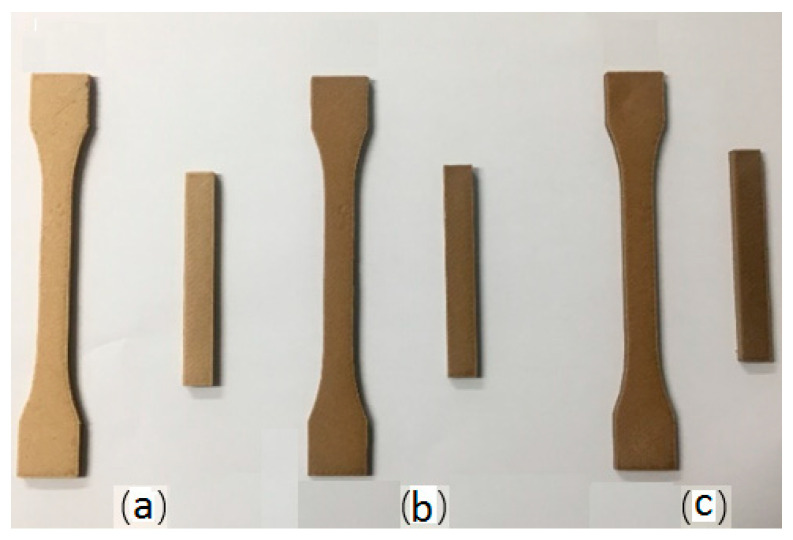
The printed samples using different natural fiber powder/PLA biocomposites: (**a**) ARP/PLA; (**b**) RSP/PLA; and (**c**) WF/PLA.

A desktop-level 3D printer (MOSHU S10, Hangzhou Shining 3D Technology Co., Ltd., Hangzhou, China) was used to print the prepared filaments into standard specimens for tensile and flexural tests, respectively. The sample model file (STL file) for the test was computer-aided designed and was then sliced and transformed into G-code. The nozzle temperature was set to 220 °C. The layer height was set to 0.1 mm, with a printing speed of 50 mm/s, a filling density of 100% and a deposition angle of 0°, and the hot bed temperature was set to 50 °C. The 3D-printed specimens are shown in Figure 2.

### 2.4. Measurement and Characterization

#### 2.4.1. Determination of Mechanical Properties

The samples were conditioned at room temperature (23 °C and 45% relative humidity) and characterized using a universal machine (E44.304, MTS Industrial Systems (China) Co., Ltd., Shenzhen, China) with a 20 kN sensor. The tensile experiments were performed in accordance with the ASTM D 638-2010 standard at a crosshead speed of 10 mm/min, and the flexural experiments were operated according to the ASTM D790-2010 standard at a crosshead speed of 5 mm/min, respectively.

#### 2.4.2. Scanning Electron Microscopy (SEM) Observation

The cross-sectional surfaces of the 3D-printed WF/PLA, ARP/PLA, and RSP/PLA specimens were observed by an SU 8010 field-emission scanning electron microscope (Hitachi Corporation, Tokyo, Japan) at an accelerating voltage of 3 kV. The observed biocomposite samples were obtained from the fractured flexural specimens. Before observation, the fracture surfaces of the composites were sputtered with a gold conductive film to avoid electrical charging during examination.

#### 2.4.3. Thermogravimetric Analysis (TGA)

A thermal gravimetric analyzer (TG 209F1, NETZSCH-Gerätebau GmbH, Selb, Germany) was used to evaluate the thermal stability of the raw materials and FDM 3D-printed specimens. Approximately 5 mg–12 mg of each sample was heated from 20 °C to 550 °C under nitrogen atmosphere at a 20 K/min heating rate.

#### 2.4.4. Differential Scanning Calorimetry Assessment

Differential scanning calorimetry (DSC214, NETZSCH-Gerätebau GmbH, Selb, Germany) was used under a nitrogen atmosphere to investigate the melting and crystallization behavior. First, approximately 5.0 mg sample was heated from the ambient temperature to 220 °C, at a heating rate of 10 °C/min and held in an isothermal state for 5 min, to remove the heat history, residual moisture, and voids. Then, the sample was cooled down to room temperature at 10 °C/min, and reheated to 220 °C at 10 °C/min. The cold-crystallization temperature (T_cc_) and the melt temperature (T_m_) were determined from the second heating curves. The NETZSCH analysis software was employed to assess the glassy transition temperature (T_g_) and heat capacity. The sample’s degree of crystallinity was calculated using the following equation [27]:

(1) $$\chi_{c}=\frac{\left|{\Delta H}_{m}+{\Delta H}_{\mathrm{cc}}\right|}{{\omega \Delta H}^{*}\;}\times 100\%$$

where ω represents the PLA mass fraction in the material, $\Delta H_{m}$ represents the observed enthalpy of fusion, $\Delta H_{\mathrm{cc}}$ represents the observed enthalpy of cold crystallization, $\Delta H^{*}$ represents the standard melting enthalpy of 100% crystallization of PLA, which was 93.7 J/g [28,29].

#### 2.4.5. Dynamic Mechanical Thermal Analysis (DMTA)

The dynamic mechanical properties of the FDM 3D-printed biocomposites were tested using Dynamic Mechanical Analyzer (DMA 242C, Netzsch, Bavaria, Germany) under a nitrogen atmosphere. The dual cantilever fixture was used to hold the specimen during the testing. The test was conducted at a sinusoidal frequency of 5.0 Hz, over a temperature range of 20~100 °C at a heating rate of 5 °C/min.

#### 2.4.6. Wettability Testing

A contact angle instrument (DSA100; KRÜSS GmbH, Borsteler Chaussee, Germany) was employed to test the contact angles of a distilled water drop on the surface of each FDM 3D-printed biocomposite sample at room temperature. A 5 µL droplet of distilled water was dropped onto the surface and kept for 15 s, and then the contact angles from the images were measured at different points, and 10 specimens were used for each sample.

## 3. Results and Discussion

### 3.1. Mechanical Properties

**Figure 3:**
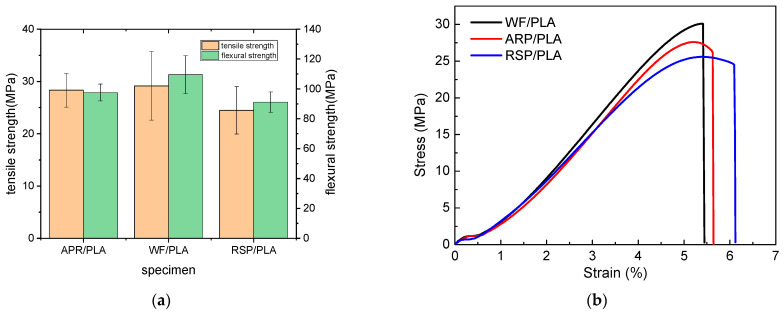
Mechanical properties of FDM 3D-printed specimens (**a**) strength; and (**b**) δ–ε curve.

The mechanical properties’ patterns of 3D printing materials are presented in Figure 3. The results show that the tensile and flexural strengths of ARP/PLA biocomposite specimens were 28.33 MPa and 97.60 MPa, respectively, which were 2.85% and 10.89% smaller than those of WF/PLA, while 15.73% and 7.04% greater than those of RSP/PLA. The stress (δ) vs. strain (ε) curves during the tensile test of the three biocomposites displayed in Figure 3b indicated that the elongation at break for each specimen falls into the range between 5.4~6.2%. For WF/PLA, no yield point existed in its δ–ε curve, showing typical brittle fracture characteristics. However, a yield point could be found at a strain of 5.2% for ARP/PLA and 5.4% for RSP/PLA, then the specimens broke at a strain of 5.7% and 6.2%, respectively, indicating that the samples of ARP/PLA and RSP/PLA biocomposites both showed ductile fracture, but not obviously. After comparison, we found that, the toughness of ARP/PLA was better than WF/PLA, but poorer than RSP/PLA. 

### 3.2. Morphological Analysis

**Figure 4:**
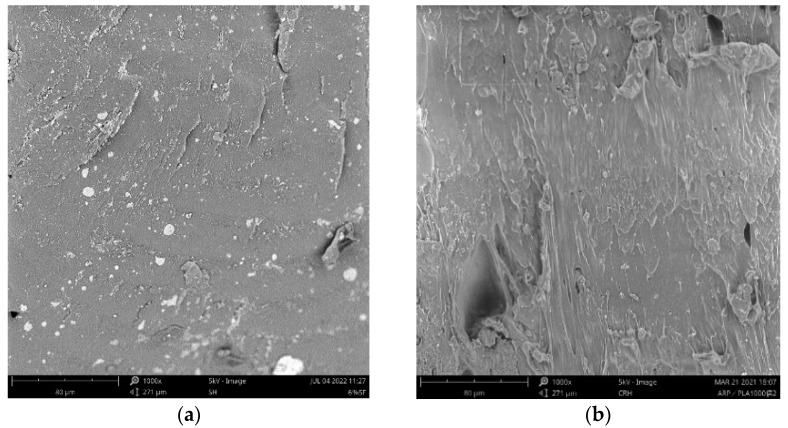

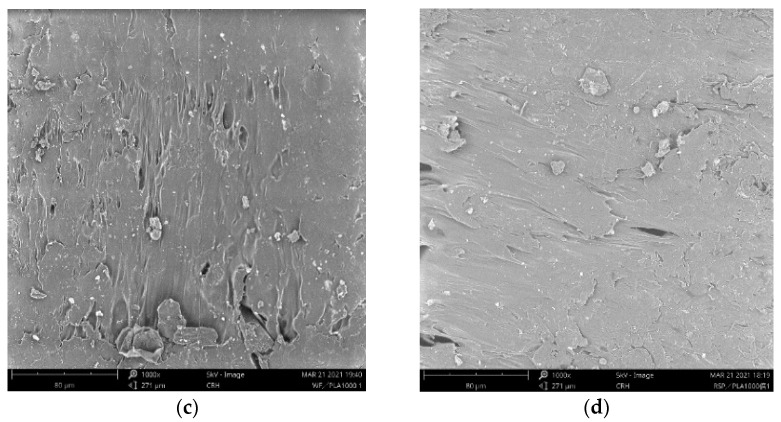
Micro morphologies of PLA and different plant fiber/PLA biocomposite specimens: (**a**) PLA; (**b**) ARP/PLA; (**c**) WF/PLA; and (**d**) RSP/PLA.

The cross-sectional morphologies of WF/PLA, RSP/PLA, and ARP/PLA biocomposites were visualized from the collected materials after the flexural testing by SEM micrographs and it was shown in Figure 4. The SEM image of the broken section of pure PLA was also illustrated in Figure 4 as a reference. The surface of pure PLA was found to be smooth and uniform and there were almost no pores and voids in the matrix, indicating that there was a close bond between the PLA matrix and the PLA was fractured brittlely. The interface of PLA was a little affected by the incorporation of natural plant powder into PLA. The fracture surface of the samples became a little rough and uneven, but no natural fibers were pulled out of the matrix. The reason may be that the natural fibers can adhere to the PLA matrix tightly after the specimen being FDM 3D-printed, when a load was exerted on the specimen, the fibers would deform simultaneously with PLA, and then they would be broken together. This meant that the addition of the natural fiber had not obviously changed the characteristics of brittle fracture of PLA, which was consistent with the results of the mechanical testing.

### 3.3. Thermal Stability

**Figure 5:**
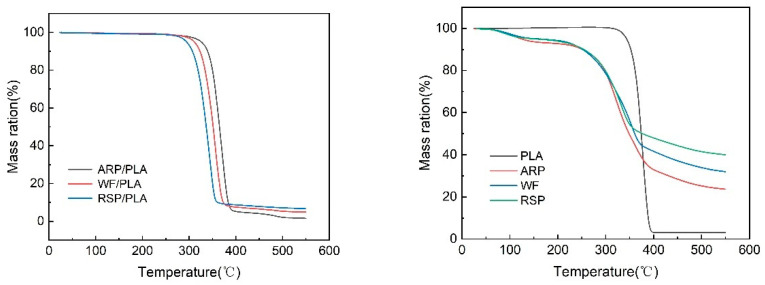
The mass loss curves of different samples.

The thermal performance of 3D printing materials reflects the thermal stability of the material. In order to comparatively investigate the effects of the incorporation of WF, RSP, or ARP on the thermal behavior of PLA, the thermal degradation properties of the 3D-printed pure PLA and the natural fiber/PLA biocomposites were studied, as shown in Figure 5; as a reference, the TGA curves of the WF, RSP, and ARP are also given in Figure 5, the thermal stability of each sample was evaluated by the temperature at which it began to decompose (T_i_) and the temperature at which it lost its weight at the maximum rate (T_p_). The corresponding temperatures and residues after thermal decomposition derived from Figure 5 are summarized in Table 1.

From Figure 5 and Table 1, the 3D-printed natural fiber/PLA biocomposites all not only began to decompose at much lower temperatures, but also had much lower T_p_ values than PLA, indicating that the fibers caused a reduction in the thermal stability of the composites. This effect was evidenced in some other literatures [28] and is consistent with the conclusion of this study. Among the composites, however, the ARP/PLA was the most thermal stable, and the RSP/PLA was the least, the T_i_ value of ARP/PLA (343.2 °C) was 15.9 °C and 31.1 °C higher than those of WF/PLA and RSP/PLA, respectively, in the meantime, its T_p_ value of 370.9 °C was 15.6 °C and 25.3 °C higher than those of the latter two composites. The thermal stability of the three printed samples was different due to the different stability of each natural fiber as shown in Figure 5b and Table 1, however, in this situation, ARP showed the most thermal stable because of its higher T_i_ and T_p_ values than RSP and WF. In addition, the three kinds of natural fibers all had much lower T_i_ and T_p_ values than PLA, as a result, the thermal stability of PLA would be worsened once ARP, WF, or RSP was incorporated into PLA.

### 3.4. Non-Isothermal Crystallization Behavior

**Figure 6:**
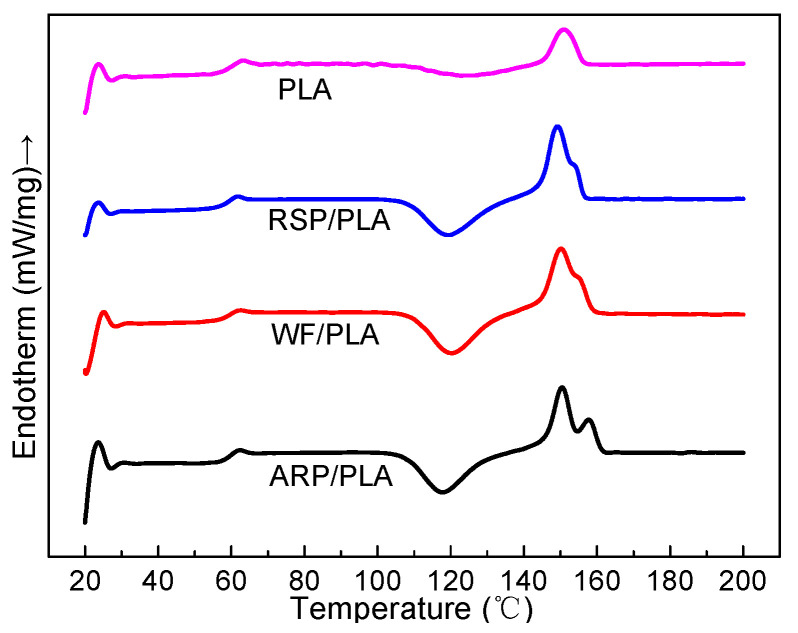
The secondary heating curves of different samples.

The melting and crystallization behavior of polymers has effects on the crystal structure, crystallinity, and end performances of the material [15]. Here, the effect of the natural fiber as filler on the crystallization and melting behavior of 3D-printed samples was studied by means of DSC. As shown in Figure 6, the second heating cycles in the DSC thermograms of samples with any processing and thermal history removed. Some information such as the glassy transition temperature (T_g_), the melting temperature (T_m_), and the crystallinity (Χ_c_) could be concluded from the DSC curves, and as demonstrated in Table 2, a big cold-crystallization peak appeared during the second heating process. This crystallization behavior was attributed to the excessively slow crystallization rate of the pure PLA to organize its molecular chains in a timely manner, and the cold-crystallization temperature obviously did not vary with the addition of ARP, WF, or RSP, which meant that the addition of the natural fiber had no significant effect on the cold-crystallization behavior of PLA. In addition to these, the T_g_ and T_m_ values of the biocomposites were all near to those of pure PLA. Thus, all the biocomposites in this study could be processed at temperatures near to that of PLA. In addition, it could also be observed from Table 2 that the crystallinity of WF/PLA or ARP/PLA was much greater than that of pure PLA, although the absolute crystallinity was not very high, which confirmed that the incorporation of natural fiber could improve the crystallization of the PLA matrix. A similar conclusion was also drawn for the sugarcane bagasse/PLA biocomposite [15].

### 3.5. Thermo-Dynamic Mechanical Properties

**Figure 7:**
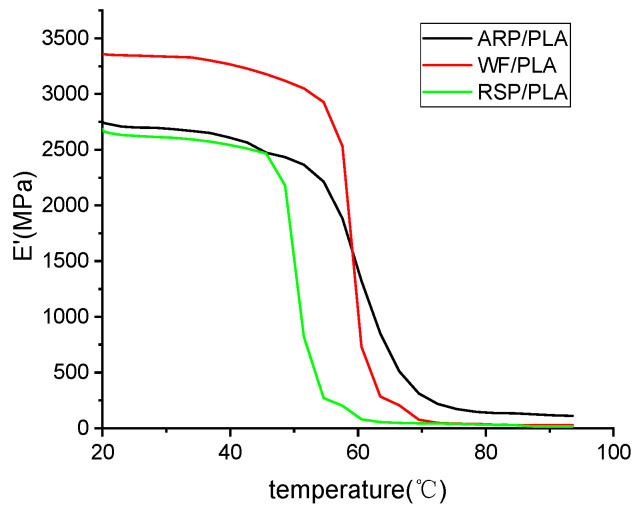
Storage modulus diagram of different samples (3.33 hz).

Figure 7 depicted the variation in the storage modulus (E’) of the developed biocomposites as a function of temperature. It was found that the storage modulus for all the biocomposites began as a plateau, and WF/PLA had the greatest storage modulus of 3361.3 MPa at the beginning, followed by ARP/PLA (2759.4 MPa) and RSP/PLA (2691.5 MPa), which was in line with the trend previously observed in the mechanical properties’ testing, indicating that the stiffness of the biocomposites could be ordered as WF/PLA > ARP/PLA > RSP/PLA. The E’ dramatically decreased at a temperature between 40 °C and 70 °C, which was due to the reduction in stiffness in the transition region [30]. However, at higher temperatures, for example, at 85 °C, the storage modulus of ARP/PLA was found to be maximum, followed by WF/PLA and RSP/PLA, respectively, which meant that the ARP/PLA was the most thermally stable at this time, and the results were in accordance with the conclusions from the TGA testing.

### 3.6. Wettability

**Figure 8:**
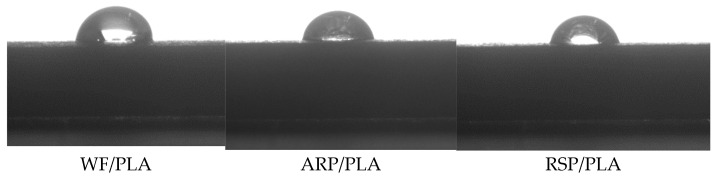
Contact angles of deionized water on surfaces of different samples.

The shapes of the water droplets on the surfaces of the printed samples are presented in Figure 8. Table 3 tabulates the measured averaged contact angles of the samples. The results show that the contact angles of WF/PLA, ARP/PLA, and RSP/PLA were 77.6°, 73.5°, and 71.2°, respectively, all of which are smaller than 90°, and thus, they were all hydrophilic. For natural fibers, ARP, WF, and RSP were all composed of cellulose, hemicellulose, lignin, and some other components, the existence of a large amount of the polar groups, such as hydroxyl in the structures of these components, made the fibers be hydrophilic. When they were complexed with PLA, the hydrophilicity of PLA was accordingly enhanced. The hydrophilicity of the three composites were ordered as RSP/PLA > ARP/PLA > WF/PLA.

## 4. Conclusions

In this study, ARP/PLA biocomposites were FDM 3D-printed, whilst WF/PLA and RSP/PLA biocomposites were prepared by the same method. Some properties of different 3D-printed specimens were comparatively investigated. Based on this research, the following conclusions can be formulated:

(1) ARP/PLA composite had moderate tensile and flexural strengths, and its tensile strength of 28.33 MPa and flexural strengths of 97.60 MPa were smaller than those of WF/PLA by 2.85% and 10.89%, respectively, despite being 15.73% and 7.04% greater than those of RSP/PLA. WF/PLA fractured brittlely, ARP/PLA and RSP/PLA both showed ductile fracture, but not obviously. No natural fibers were found to be pulled out of the matrix after breaking for any composite.

(2) The thermal stability of ARP is the best and RSP was the worst, accordingly, ARP/PLA were the most thermal stable, followed by WF/PLA and RSP/PLA in turn.

(3) The incorporation of natural plant powder increased the crystallinity of PLA, but had no significant effect on the cold-crystallization behavior of the resin. All the biocomposites could be processed at temperatures near to that of PLA because of the closeness in their proximate glassy transition temperatures (T_g_) and melting temperatures (T_m_).

(4) The storage modulus of WF/PLA, ARP/PLA, and RSP/PLA at 20 °C was 3361.3 MPa, 2759.4 MPa, and 2691.5 MPa, respectively. However, at temperatures above T_g_, the storage modulus of ARP/PLA became the greatest one, followed by WF/PLA and RSP/PLA.

(5) All the biocomposites were hydrophilic and the contact angles of WF/PLA, ARP/PLA, and RSP/PLA were 77.6°, 73.5°, and 71.2°, respectively.

In summary, ARP is an effective filler for FDM 3D-printed ARP/PLA biocomposites in terms of mechanical, thermal, dynamic mechanical thermal properties, and wettability. Just like WF/PLA and RSP/PLA, ARP/PLA could not only be applicable to 3D printing, but also be environmentally friendly and promising in the field of printing.

## Figures and Tables

**Table 1:** Thermogravimetric analysis information of different samples.

Specimen	T_i_/°C	T_p_/°C	W/% (550 °C)
PLA	355.7	378.1	2.97
ARP/PLA	343.2	370.9	1.55
WF/PLA	327.3	355.3	4.89
RSP/PLA	312.1	345.6	6.68
ARP	291.6	354.8	40.0
WF	283.3	332.1	31.9
RSP	268.8	323.1	23.6

**Table 2:** DSC thermal information of different samples.

Specimen	T_g_/°C	T_cc_/°C	T_m_/°C	ΔH_cc_/(J/g)	ΔH_m_/(J/g)	Χ_c_/%
PLA	63.2	121.8	151.3	−10.75	11.06	0.3
ARP/PLA	62.4	117.8	150.5	−27.58	29.19	2.0
WF/PLA	62.4	120.4	150.2	−27.68	29.27	1.9
RSP/PLA	61.7	119.1	149.3	−27.76	27.49	0.3

**Table 3:** Contact angles of deionized water on surfaces of different samples.

Specimen	RSP/PLA	ARP/PLA	WF/PLA
Contact angle/°	71.2 ± 0.7	73.5 ± 0.5	77.6 ± 0.6

## Data Availability

Not applicable.

## Sample Availability

Samples of the compounds are not available from the authors.
